# Supplementary material for: IL-1β-Induced CXCL10 Expression in THP-1 Monocytic Cells Involves the JNK/c-Jun and NF-κB-Mediated Signaling
Source: Pharmaceuticals (Basel). 2024 Jun 22;17(7):823. doi: 10.3390/ph17070823 (PMC11279630; doi:10.3390/ph17070823)
Supplement: Supplementary file 1 [file pharmaceuticals-17-00823-s001.zip › pharmaceuticals-3004066-supplementary/pharmaceuticals-3004066-supplementary.pdf]

Supplementary Figure S1

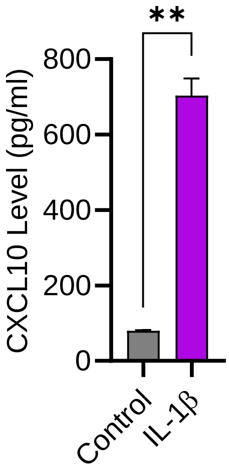

Figure S1. IL-1β stimulation induces CXCL10 production in THP-1 derived macrophages. THP-1 cells were cultured (1×10^6 cells per well) in 12-well plates and cells were incubated for 3 days with PMA (10 ng/mL). After 3 days, medium was replaced with fresh medium and kept the macrophages in resting phage for 24 hrs. Macrophages were treated with IL-1β or vehicle (control) for 24 hrs. CXCL10 secreted protein in culture supernatants was determined using ELISA. All data are expressed as mean ± SEM values (n = 3; **$p \leq 0.001$).
